# Supplementary figures and images for: Thymoquinone Antifungal Activity against Candida glabrata Oral Isolates from Patients in Intensive Care Units—An In Vitro Study
Source: Metabolites. 2023 Apr 21;13(4):580. doi: 10.3390/metabo13040580 (PMC10143056; doi:10.3390/metabo13040580)

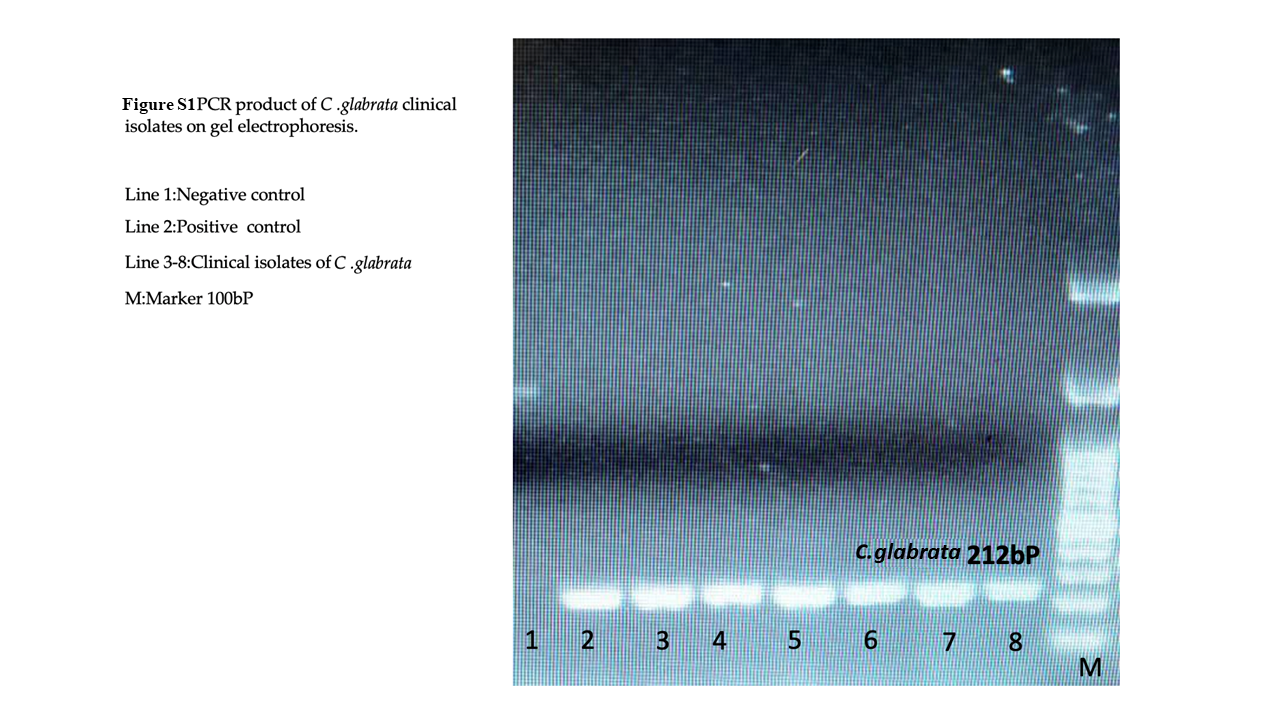

Supplement: Supplementary file 1 [file metabolites-13-00580-s001.zip › metabolites-2242256-supplementary figure S1.png]
